# Analysis of Codon Usage Bias in Chloroplast Genomes of *Dryas octopetala* var. *asiatica* (Rosaceae)

**DOI:** 10.3390/genes15070899

**Published:** 2024-07-09

**Authors:** Lizhen Ling, Shudong Zhang, Tao Yang

**Affiliations:** Key Laboratory for Specialty Agricultural Germplasm Resources Development and Utilization of Guizhou Province, Liupanshui Normal University, Liupanshui 553004, China; primula_ling@foxmail.com (L.L.); 19885348955@163.com (T.Y.)

**Keywords:** *D. octopetala* var. *asiatica*, chloroplast genome, codon usage bias, optimal codon

## Abstract

*Dryas octopetala* var. *asiatica*, a dwarf shrub belonging to the Rosaceae family and native to Asia, exhibits notable plasticity in photosynthesis in response to temperature variations. However, the codon usage patterns and factors influencing them in the chloroplast genome of this species have not yet been documented. This study sequenced and assembled the complete genome of *D. octopetala* var. *asiatica*. The annotated genes in the chloroplast genome were analyzed for codon composition through multivariate statistical methods including a neutrality plot, a parity rule 2 (PR2) bias plot, and an effective number of codons (ENC) plot using CodonW 1.4.2 software. The results indicated that the mean GC content of 53 CDSs was 38.08%, with the average GC content at the third codon base position being 27.80%, suggesting a preference for A/U(T) at the third codon position in chloroplast genes. Additionally, the chloroplast genes exhibited a weak overall codon usage bias (CUB) based on ENC values and other indicators. Correlation analysis showed a significant negative correlation between ENC value and GC2, an extremely positive correlation with GC3, but no correlation with GC1 content. These findings highlight the importance of the codon composition at the third position in influencing codon usage bias. Furthermore, our analysis indicated that the CUB of the chloroplast genome of *D. octopetala* var. *asiatica* was primarily influenced by natural selection and other factors. Finally, this study identified UCA, CCU, GCU, AAU, GAU, and GGU as the optimal codons. These results offer a foundational understanding for genetic modification and evolutionary dynamics of the chloroplast genome of *D. octopetala* var. *asiatica*.

## 1. Introduction

The genetic material of DNA typically transmits genetic information to proteins through RNA in the form of triplets. In the genetic code, there are 64 standard codons, including UAA, UAG, and UGA translation termination signal codons, and the other 61 codons encode 20 common amino acids. Among these amino acids, methionine and tryptophan are encoded by one codon, AUG and UGG, respectively, and the other 18 amino acids are encoded by at least two codons. The degeneracy of the genetic code allows the same amino acid to be encoded by multiple different codons, termed as synonymous codons [1]. However, these synonymous codons are not utilized equally, leading to codon usage bias (CUB). Significant variations in the usage of synonymous codons have been observed among plant genes and different organisms [2]. For example, a gradient of GC content and codon usage exists in correlation with the direction of transcription in Graminae genes. Monocot genes show up to 25% higher GC content at their 5′ ends compared to their 3′ ends, a pattern not observed in dicot genes [3]. Codon bias plays a crucial role in various cellular processes, such as transcription, mRNA stability, translation efficiency and accuracy, and protein expression and co-translational folding [4,5,6]. The codon preference is often affected by many factors such as environmental conditions, genetic drift, base mutations, genome size, tRNA abundance, and gene expression levels [7,8].

Chloroplasts (cps) are crucial organelles within plant cells, serving essential functions in photosynthesis, metabolic regulation, and signal transduction. These organelles possess distinct structural and compositional features, as well as a remarkable ability to adjust and thrive in response to diverse environmental factors. Chloroplasts have their own genomes and can carry out some metabolic reactions independently. In land plants, the chloroplast genome size typically ranges from 120 to 180 kb, varying in specific sizes among species. The chloroplast genome encompasses critical genes for photosynthesis and plays an important role in plant evolution [9]. The chloroplast genome in land plants originates from endophytic cyanobacteria and typically features a circular structure, consisting of two inverted repeats (IRs), a large single copy region (LSC), and a small single copy region (SSC) [9,10]. Studies have shown that the chloroplast genome of land plants contains approximately 110–130 different genes, with over 80 coding genes primarily involved in photosynthesis or metabolic processes [9]. Therefore, a comprehensive knowledge of plant chloroplast genomes can aid researchers in understanding the expression of functional proteins, thereby contributing to advancements in genetics and molecular biology. The advancement of high-throughput sequencing technology and reduced sequencing costs have enabled the sequencing of chloroplast genomes in numerous plant species. Codon preferences in multiple plant chloroplast genomes, such as *Gynostemma pentaphylla* [11], *Trichoderma pentaphylla* [12], and *Lespedeza* [13], have been analyzed and documented.

*Dryas octopetala*, a dwarf shrub in the Rosaceae family, thrives in the alpine and Arctic regions of the Northern Hemisphere. The living environment of *D. octopetala* is extremely harsh, with a damp climate, low temperature, and thin soil layers with little nutrients. Therefore, *D. octopetala* has a short growth cycle and slow growth rate. In the long-term evolutionary process, *D. octopetala* has developed special abilities to adapt to the harsh polar environment. Studies have reported that it can acquire essential nutrients like nitrogen and phosphorus through a symbiotic relationship with ectomycorrhizae (ECM) for their own growth [14,15,16,17]. At present, *D. octopetala* has become a key player in studying the role of fungi in community ecology [14]. In addition, *D. octopetala* is a living fossil that records climate change and is very important in studying it [18,19]. There are two varieties of *D. octopetala*: the original *D. octopetala* L. var. *octopetala* and the unique *D. octopetala* var. *asiatica* sporadically found in alpine tundra regions in the northeast of Asia. Recent studies have shown that the photosynthesis of *D. octopetala* var. *asiatica* is very sensitive to climate warming, demonstrating relatively strong plasticity to temperature increases [20]. To better understand *D. octopetala* var. *asiatica*, this research specifically investigated the codon usage bias of the genes within the chloroplast genome, clarifying the main factors influencing this bias in this species. This study is significant for understanding the molecular evolution of chloroplast genes and optimizing the incorporation of foreign genes in *D. octopetala* var. *asiatica*.

## 2. Materials and Methods

### 2.1. Sequence of Chloroplast Genome of D. octopetala var. asiatica

The leaves of *D. octopetala* var. *asiatica* were collected from Changbai Mountain in Jilin province, China, using standard botanical collection methods, and the voucher specimen was deposited in the Herbarium of Kunming Institute of Botany, Chinese Academy of Sciences. Genomic DNA was extracted from silica gel-dried leaves using a modified CTAB method [21], and the constructed library was used to be sequenced on the Illumina NovaSeq PE150 platform (Illumina, Inc., San Diego, CA, USA) following the manufacturer’s protocols. The assembly and annotation of the chloroplast genome of this species were conducted as previously described [22]. The annotated chloroplast genome information of *D. octopetala* var. *asiatica* was submitted to the NCBI GenBank database with the accession number OQ420424.

### 2.2. Analysis of Codon Usage Characteristics

The coding protein sequences (CDSs) were extracted from the *D. octopetala* var. *asiatica* chloroplast genome according to the annotation information. The CDSs of genes were removed for analysis based on the following criteria: (1) genes contained the stop codons in the CDS (i.e., pseudogenes); (2) genes were duplicated in chloroplast genome (i.e., duplicated genes in the IR regions); (3) the gene sequences were less than 300 bp in length (gene length < 300 bp). The remaining CDSs were selected from the chloroplast genome and analyzed for subsequent analysis.

CodonW 1.4.2 software was used to calculate the occurrence frequency of GC1, GC2, and GC3 content and the average GC (GCall) base content. Codon usage bias was often assessed using relative synonymous codon usage (RSCU) and effective codon number (ENC) with the CodonW 1.4.2 software [23,24]. ENC provides an indication of the codon bias strength, with values ranging between 20 and 61, where ENC values ≤ 35 suggest significant codon bias within genes. Conversely, a higher ENC value indicates weaker codon usage bias [25]. The RSCU value was computed to assess the bias in codon usage among genes by comparing the observed frequency of a synonymous codon with its expected frequency. It is derived using the following formula:

$$RSCU=\frac{Xij}{\sum_{j}^{ni}Xij}ni$$

Xij represents the frequency of codon i encoding the j-th amino acid, while ni refers to the number of codons encoding the i-th amino acid. In the absence of codon usage bias, the RSCU value for the codon would be 1. An RSCU value greater than 1 for a specific codon indicates that this codon is being used at a relatively higher frequency [26,27].

### 2.3. Analysis of the Source of Codon Usage Bias

A neutral plot was used to scrutinize the impact of mutation pressure and natural selection on codon usage. A regression line was drawn with GC3 as the x-coordinate and the average of GC1 and GC2 (GC12) as the y-coordinate. When the slope of the regression curve is close to 0, this suggests that natural selection plays a key role in shaping codon usage. When the slope is near 1, this implies that the base composition at positions 1, 2, and 3 of GC codons is similar and codon bias is mainly influenced by mutation [28].

Parity rule 2 (PR2) bias plot analysis is utilized to assess the impact of natural selection and mutation pressure on the third codon position. The composition of the four bases A, T, C, and G at the third codon position of each gene was calculated to obtain GC bias [G3/(G3 + C3)] and AU bias [A3/(A3 + T3)]. A plot was generated by plotting AU bias against GC bias, illustrating the correlation between the purine (A and G) and pyrimidine (C and T) content in genes. The central point in the plot represents the equilibrium state (A = T, C = G), where both coordinates are 0.5. The distance and direction of a gene site from this central point indicate the degree of deviation from PR2 [29]. When more genes cluster near the standard curve, it suggests that the codon usage preference is likely to be entirely caused by mutation, whereas a deviation from the curve indicates a stronger role of natural selection in the codon usage.

ENC reflects the degree of codon dispersal and is widely used as an indicator to measure codon preference. The ENC plot was generated with ENC values on the y-axis and GC3 on the x-axis. This is a two-dimensional scatter plot that includes a reference line representing the expected values. By analyzing the distance between the actual ENC values and the expected values for various functional genes, we can observe the relationship between codon preference and different functional genes [30]. When the scatter points are close to the reference line, it indicates that codon preference is mainly influenced by mutation, while a greater distance suggests a stronger influence of natural selection. The standard curve used in this study was obtained from the equation ENCexp = 2 + GC3s + 29/[GC3s2 + (1 − GC3s)2], which represents the expected ENC value [29].

### 2.4. Identification of Optimal Codons

The 53 CDSs that met the criteria were ranked based on their ENC values. The top and bottom 5% of the ranking were selected to establish the high-expression gene (HEG) library and low-expression gene (LEG) library, respectively. Subsequently, the RSCU values of the high- and low-expression libraries were calculated, and the difference between the high- and low-expression libraries was determined, denoted as △RSCU. In the gene library, codons with ΔRSCU > 0.08 were identified as high-expression advantageous codons [31]. The optimal codons are determined by combining codons with ΔRSCU > 0.08 and RSCU > 1, combining high-expression advantageous codons with high-frequency codons [31].

## 3. Results

### 3.1. Annotation and Analysis of Protein-Coding Genes

The complete chloroplast genome of *D. octopetala* var. *asiatica* was assembled based on high-throughput sequencing data. A total of 84 protein-coding genes (PCGs) were annotated in the cp genome of this species. After filtering out genes with sequence lengths shorter than 300 bp, duplicated genes, and pseudogenes, 53 PCGs were selected for codon analysis. These genes were mainly categorized into three functional classifications, including photosynthetic, self-replication, and other functional groups (Table 1). Of them, eight genes (*petB*, *petD*, *atpF*, *ndhA*, *ndhB*, *rpoC*, *rpl2* and *rpl16*) contained one intron, whereas the remaining genes were all coding sequences (Table 1).

### 3.2. Analysis of Codon Usage Bias

**Figure 1:**
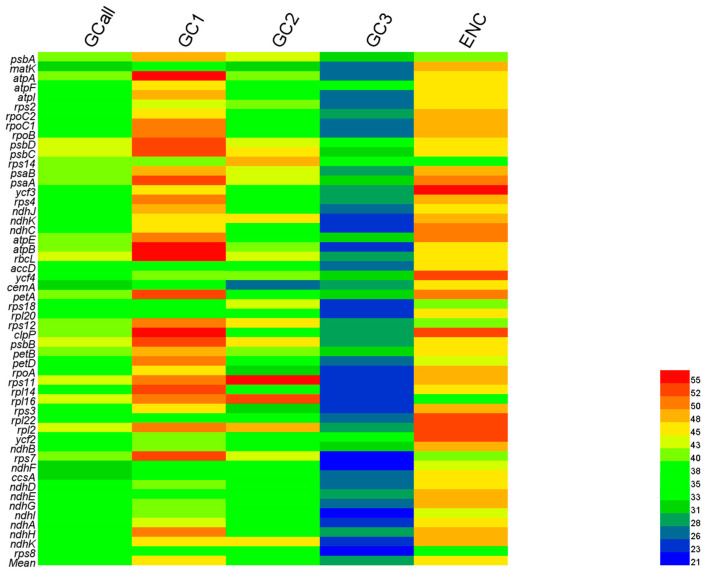
Heat map of GC contents and ENC of each *D. octopetala* var. *asiatica* chloroplast gene.

In this study, codon bias was primarily evaluated by analyzing GC content, the effective number of codons (ENC), and relative synonymous codon usage (RSCU). In the *D. octopetala* var. *asiatica* chloroplast genome, the GC contents of 53 genes (GCall) ranged from 29.4% to 44.1%, with an average of 38.08% (Figure 1). Further analysis of the three positions of codons (GC1, GC2, and GC3) in these genes revealed GC contents ranging from 34.47% to 57.77% for GC1, 27.39% to 56.12% for GC2, and 21.43% to 36.78% for GC3 (Table 2). On average, the GC contents of these three positions were 46.80%, 39.43%, and 28.03%, respectively (Figure 1). In addition, the frequencies of four nucleotides on the third position of codons were as follows: U(T)3s (35.19~59.49%) > A3s (29.76~56.25%) > G3s (7.66~26.15%) > C3s (9.00~25.00%). The expression levels of U(T) and A were higher compared to G and C (Table S1). These results show an uneven distribution of the four bases across the three codon positions, with an overall preference for A/U(T) bases. Notably, a lower GC content was observed at the third nucleotide position in codons, suggesting a bias towards A or U(T) at the third codon position.

The ENC values of 53 genes ranged from 33.14 to 57.39, with an average of 47.20 (Figure 1). A higher ENC value indicates a weaker codon bias, and an ENC value ≤ 35 is indicative of genes with significant codon bias [29]. Therefore, *D. octopetala* var. *asiatica* exhibited a weak codon preference. Moreover, analysis of RSCU values showed CUU as the most frequent codon (RSCU = 2.02) and CUC as the least frequent (RSCU = 0.36) (Table 2). There were 31 high-frequency codons with RSCU values greater than 1, predominantly ending in A/U, while codons with RSCU values less than 1 were mainly ending in G/C (Figure 1). Overall, these results indicated that the cp genome of *D. octopetala* var. *asiatica* was rich in A/U bases and exhibited a preference for codons ending with A/U in this species.

Our study delved deeper into the correlation between GCall and GC1, GC2, and GC3. The results indicated a strong positive correlation between GCall and the GC content at all three positions (GC1, GC2, and GC3) (Table 3). Specifically, a very significant correlation was observed between GC1 and GC2, while a significant correlation was found between GC1 and GC3 (Table 3). On the other hand, the correlation between GC2 and GC3 was not deemed significant (Table 3). These findings suggested that the base compositions at the first codon position had a higher similarity to those of the second position than the third position, with a notable difference in base composition between the second and third positions. Additionally, it was noted that ENC did not show a correlation with GCall and GC1 (*p* < 0.05). However, ENC displayed a significant negative correlation with GC2 content and a very significant positive correlation with GC3 content (Table 3). Moreover, the size of codons did not exhibit any correlation with GC1, GC2, GC3, GCall, or ENC. These results highlighted that the GC content of codons did not vary based on the size of codons. Furthermore, the GC compositions at the first position of the codon were linked to those at the second and third positions, while the GC compositions at the third position did not show any relation to the GC content at the second position.

### 3.3. Analysis of the Source of Codon Usage Bias

**Figure 2:**
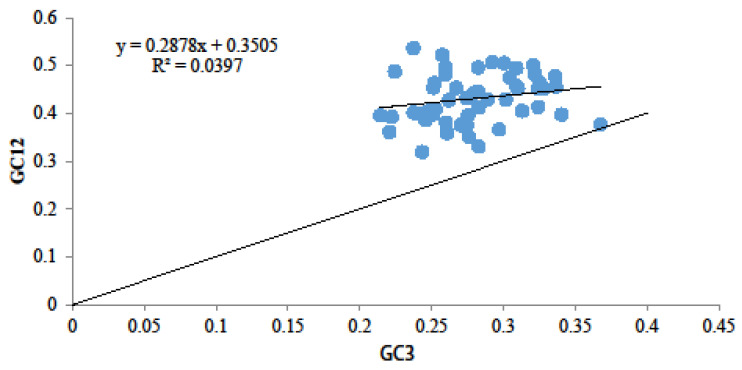
Neutral plot analysis of chloroplast codons of *D. octopetala* var. *asiatica*. Note: The slope curve shows that GC12 is equal to GC3. Different coding genes are marked by blue colors.

A neutral plot was used to examine the relationship between GC12 and GC3 to assess the impact of mutation and natural selection on codon preference. The results indicated that GC12 had a distribution range of 0.3–0.6, while GC3 ranged from 0.2 to 0.4 (Figure 2). Pearson correlation analysis demonstrated a weak correlation between GC12 and GC3, with a regression coefficient (R2) of 0.039 across all coding genes (Figure 2). Additionally, most genes were situated above the diagonal line, indicating a deviation of the correlation coefficient value from 1. These results imply that natural selection has a more substantial influence than mutation on codon preference in the *D. octopetala* var. *asiatica* chloroplast genome.

**Figure 3:**
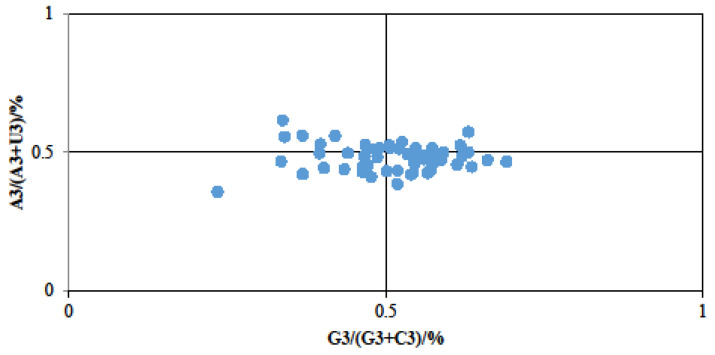
PR2 plot bias analysis of chloroplast codons of *D. octopetala* var. *asiatica*. Genes were plotted based on their GC bias [G3/(G3 + C3)] and AU bias [A3/(A3 + U3)] in the third codon position.

The impact of mutation and selection pressure on gene codon usage bias was further examined through PR2 plots. The analysis revealed that 53 genes exhibited uneven distribution across the four regions, with most genes being distant from the central region of 0.5, except for a few near the center (Figure 3). This observation suggests that natural selection might play a significant role in influencing the utilization of the third base of these gene codons. Moreover, the A3/(A3 + T3) ratio for most codon bases was below 0.5, while the G3/(G3 + C3) ratio for more than half of the gene codon bases exceeded 0.5 (Figure 3). These findings revealed that the frequency of codon base usage, particularly the third codon base, lean towards T(U) over A and G over C. Overall, the codon usage pattern of *D. octopetala* var. *asiatica* chloroplast genes was also affected by other factors such as mutations apart from natural selection.

**Figure 4:**
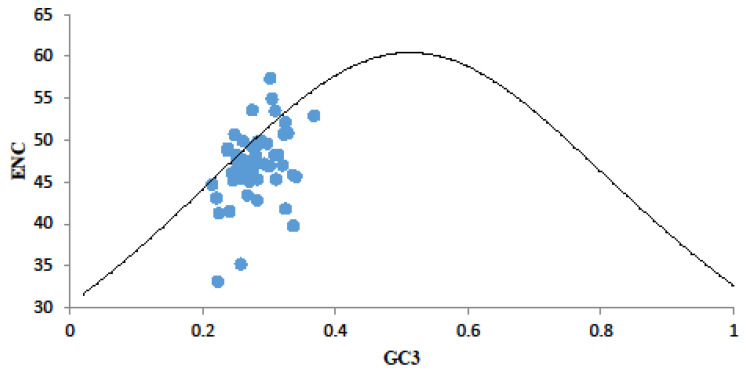
ENC plot analysis of chloroplast codons of *D. octopetala* var. *asiatica*. Note: The curve shows the relationship between ENC values and GC3S under random codon usage assumption. Different coding genes are marked by blue colors.

To evaluate codon usage preference in the chloroplast genes of *D. octopetala* var. *asiatica*, we calculated ENC values and examined their frequency distribution. Our results showed that ENC values ranged from −0.09 to 0.29, with 23 genes (43%) having an ENC value below 0.05 (Figure 4). By analyzing the ENC plot, we observed that most genes fell below the standard curve, while a few genes were scattered near it, indicating diverse codon preferences across genes. The discrepancies between expected and actual ENC value distributions suggested that natural selection played a significant role in shaping codon preference in the chloroplast genes of *D. octopetala* var. *asiatica*.

### 3.4. Identification of Optimal Codons

In this study, optimal codons were identified by analyzing ΔRSCU values in high- and low-expression genes. A total of 23 codons with values exceeding 0.08 were found in high-expression genes. By combining with 31 high-frequency codons (Table 2), 6 of 23 codons were determined as optimal codons and included UCA, CCU, GCU, AAU, GAU, and GGU (Table 4). Notably, five out of the six optimal codons ended with U and one optimal codon ended with A, which was consistent with the RSCU result.

## 4. Discussion

Synonymous codon usage preference is a common phenomenon in the genomes of various organisms. The frequency of codon usage can differ between species and even genes within the same species [32]. Although synonymous mutations at the third base of codons do not alter the amino acid, they play a significant role in determining amino acid types. Consequently, GC3 is often utilized as a crucial indicator of codon bias [33]. Our results indicated that the chloroplast genome of *D. octopetala* var. *asiatica* had an average GC content of 38.08%, suggesting a preference for codons ending in A/U(T). This pattern is also observed in numerous plant chloroplast genomes, such as *Camellia* [34] and species from the Euphorbiaceae family [35]. Moreover, the order of GC content at the three codon positions typically follows GC1 > GC2 > GC3, consistent with patterns found in most angiosperms [29].

The neutral theory of molecular evolution suggests that base mutations and natural selection have neutral or nearly neutral effects on changes in the third base of codons. When codon usage is shaped by natural selection, GC3 values typically fall within a narrow range, with no significant correlation between GC12 and GC3 [36]. The results of the neutral analysis in this study suggest that codon bias in the chloroplast genome of *D. octopetala* var. *asiatica* is primarily driven by natural selection, possibly as an adaptation to the harsh alpine environments where this species thrives. This base preference could potentially help researchers identify genetic markers or traits that confer resilience against stressors related to changing climate conditions, such as increased temperatures, by unraveling the genetic code and its functions in this species. In addition, PR2 and ENC plot analyses indicated that codon bias in the chloroplast genome of this species may also be influenced by other factors, including mutations apart from natural selection. In the case of *Delphinium grandiflorum* L., it is proposed that codons ending with A/T are predominantly shaped by natural selection [37]. Therefore, the codon bias in plants can be affected by various factors, which may differ among species.

The identification of optimal codons in the chloroplast genome of *D. octopetala* var. *asiatica* is influenced by strong positive selection or mutation. Studies have shown that codon usage patterns play a role in gene expression, with higher expression levels correlating with stronger codon preferences. This preference impacts gene regulation by influencing the accuracy and efficiency of translation [8,38]. Plant chloroplast gene expression vectors are typically designed using optimal codons to enhance gene expression. Furthermore, analyzing codon usage patterns can provide insights into the expression of genes with unknown functions [39]. Therefore, the investigation of optimal codons and codon usage patterns in this study is crucial for understanding species evolution and maximizing the expression of foreign genes in *D. octopetala* var. *asiatica*.

## 5. Conclusions

In this study, the codons in the chloroplast genome of *D. octopetala* var. *asiatica* were comprehensively analyzed for the first time. The results showed that the gene base compositions did not mainly consist of G and C, but instead had an average of 38.08%. Specifically, the GC content at the third codon position was only 27.08%, suggesting a preference for ending with A/U(T) in chloroplast genes of this species. Further analysis indicated that the primary factor influencing the codon usage preference was natural selection, with mutation also playing a role during evolution. Finally, this study identified UCA, CCU, GCU, AAU, GAU, and GGU as the optimal codons. In this study, the identified optimal codons and codon usage biases could potentially serve as targets for genetic engineering strategies aimed at enhancing the tolerance of *D. octopetala* var. *asiatica* to climate change-related stressors (i.e., increased temperatures), thereby contributing to conservation efforts and supporting its survival in the face of environmental change. Therefore, these findings provided a foundational dataset for future studies on species evolution and chloroplast genetic engineering in *D. octopetala* var. *asiatica*, particularly in relation to adapting to climate changes.

## Figures and Tables

**Table 1:** The functional categories of chloroplast genes in *Dryas octopetala* var. *asiatica.*

Classification	Gene Group	Gene Name
Genes related to photosynthesis	Photosystem I	*psaA*, *psaB*, *psaC*
	Photosystem II	*psbA*, *psbB*, *psbC*, *psbD*,
	Cytochrome b/f complex	*petA*, *petB* ^b^, *petD* ^b^
	ATP synthase	*atpA*, *atpB*, *atpE*, *atpF* ^b^, *atpI*
	NADH-dehydrogenase	*ndhA* ^b^, *ndhB* ^ab^, *ndhC*, *ndhD*, *ndhE*, *ndhF*, *ndhG*, *ndhH*, *ndhI*, *ndhJ*, *ndhK*
	Subunit of rubisco	*rbcL*
Self-replication genes	RNA polymerase	*rpoA*, *rpoB*, *rpoC1* ^b^, *rpoC2*
	Small subunit of ribosome	*rps2*, *rps3*, *rps4*, *rps7* ^a^, *rps8*, *rps11*, *rps12* ^ac^, *rps14*, *rps18*,
	Large subunit of ribosome	*rpl2* ^ab^, *rpl14*, *rpl16* ^b^, *rpl20*, *rpl22*,
Other genes	C-type cytochrome synthesis gene	*ccsA*
	Subunits of Acetyl-CoA-carboxylase	*accD*
	Envelope membrane protein	*cemA*
	Protease	*ClpP* ^c^
Unknown genes	Conserved open reading frames	*ycf1*, *ycf2* ^a^, *ycf3* ^c^, *ycf4*

Note: ^a^ indicates the double-copy gene, ^b^ indicates genes with one intron, and ^c^ indicates genes with two introns.

**Table 2:** RSCU analysis of each amino acid in *D. octopetala* var. *asiatica* chloroplast genes.

Amino Acid	Codon	Number	RSCU	Amino Acid	Codon	Number	RSCU
Phe	UUU	796	1.34	Tyr	UAU	657	1.61
	UUC	392	0.66		UAC	161	0.39
Leu	UUA	748	2.02	His	CAU	398	1.53
	UUG	460	1.24		CAC	122	0.47
	CUU	465	1.26	Gln	CAA	585	1.53
	CUC	134	0.36		CAG	180	0.47
	CUA	277	0.75	Asn	AAU	796	1.53
	CUG	139	0.38		AAC	244	0.47
Arg	AGA	394	1.87	Lys	AAA	852	1.54
	AGG	123	0.58		AAG	257	0.46
Met	AUG	490	1.00	Asp	GAU	706	1.61
Val	GUU	437	1.47		GAC	173	0.39
	GUC	125	0.42	Glu	GAA	870	1.51
	GUA	468	1.57		GAG	284	0.49
	GUG	160	0.54	Cys	UGU	178	1.52
Ser	UCU	458	1.74		UGC	56	0.48
	UCC	234	0.89	Trp	UGG	378	1.00
	UCA	311	1.18	Arg	CGU	296	1.41
	UCG	144	0.55		CGC	88	0.42
Pro	CCU	355	1.61		CGA	280	1.33
	CCC	159	0.72		CGG	83	0.39
	CCA	252	1.14	Ser	AGU	329	1.25
	CCG	118	0.53		AGC	100	0.38
Thr	ACU	442	1.63	Gly	GGU	493	1.33
	ACC	188	0.69		GGC	160	0.43
	ACA	344	1.27		GGA	586	1.58
	ACG	109	0.40		GGG	244	0.66
Ala	GCU	536	1.85	Ile	AUU	929	1.49
	GCC	173	0.60		AUC	338	0.54
	GCA	328	1.13		AUA	603	0.97
	GCG	125	0.43				

**Table 3:** Correlation analysis of codon parameters in *D. octopetala* var. *asiatica* chloroplast genes.

	GC1	GC2	GC3	GCall	ENC
GC2	0.43 **				
GC3	0.24 *	0.09			
GCall	0.84 **	0.77 **	0.49 **		
ENC	0.15	−0.30 *	0.37 **	0.04	
N	−0.14	−0.27 *	0.21 *	−0.15	0.18

Note: The asterisk (*) signifies a statistically significant correlation at the 0.05 level (*p* < 0.05), while the double asterisk (**) denotes an extremely significant correlation at the 0.01 level (*p* < 0.01).

**Table 4:** Optimal codons in the chloroplast genome of *D. octopetala* var. *asiatica.*

No.	Codon	Amino Acid
1	UCA ***	Ser
2	CCU ***	Pro
3	GCU *	Ala
4	AAU	Asn
5	GAU ***	Asp
6	GGU	Gly

Note: * indicates ∆RSCU ≥ 0.1; *** indicates ∆RSCU ≥ 0.5.

## Data Availability

The dataset generated and analyzed during the current study is available in the NCBI GenBank database (https://www.ncbi.nlm.nih.gov/genbank/, accessed on 26 March 2023) and the accession number is OQ420424.

## Supplementary Materials

The following supporting information can be downloaded at https://www.mdpi.com/article/10.3390/genes15070899/s1: Table S1: GC contents of four nucleotides on the third position of codons in each gene.
